# Efficacy of a cultured conditioned medium of exfoliated deciduous dental pulp stem cells in erectile dysfunction patients

**DOI:** 10.1111/jcmm.17072

**Published:** 2021-11-29

**Authors:** Shoji Koga, Yutaka Horiguchi

**Affiliations:** ^1^ Ginza Solaria Clinic Panagy Co., Ltd. Tokyo Japan; ^2^ Department of Urology Edogawa Hospital Tokyo Japan

**Keywords:** erectile dysfunction, exfoliated deciduous dental pulp, International Index of Erectile Function, stem cell‐derived growth factor, vascular endothelial cells

## Abstract

Majority of current treatment strategies against erectile dysfunction (ED) has been consisted of only a supportive care to sustain enough erection during a sexual intercourse. In this study, we investigated whether the cultured conditioned medium of human exfoliated deciduous dental pulp stem cells (SHED‐CM) had an ability to treat ED through fundamentally repairing the pathological damage of vascular endothelial cells of the corpus cavernosum. An open‐label pilot study was performed from April 2016 to October 2020. SHED‐CM was injected directly into the corpus cavernosum of penis of 38 ED patients who visited our clinic and fulfilled the inclusion criteria. Efficacy was assessed using the simplified International Index of Erectile Function (IIEF‐5) questionnaire. The average age and initial IIEF‐5 score of the patients enrolled in this study was 56 (31–79) years old and 13.1 (5–20) points, respectively. Medical history revealed 7 patients with diabetes, 7 patients with hypertension and 1 patient with priapism undergone shunt operation. Of these, 37 patients (97.4%) showed an improvement in IIEF‐5 of an average of 19.3 (7–25) points or 64.4 (10–300) % increase after three injections of SHED‐CM. Eighteen patients (47.4%) achieved more than 21 points (no ED) in IIEF‐5. No adverse events were encountered. This is the first clinical report of ED treatment in the literatures evaluating the efficacy of SHED‐CM. Treatment with SHED‐CM is expected to repair vascular damages of the corpus cavernosum, which are the main cause of ED, and to be widely spread as a fundamental clinical application for ED.

## 1 INTRODUCTION

Erectile dysfunction (ED) is widely known to increase with ageing.^1^ It has been estimated that 1 in 3 Japanese men over 40 years old has ED due to the diversification of lifestyle‐related diseases and the increase in social stress.^2^ Thus, the number of ED patients in Japan has been estimated to be 11.3 million.^3^ According to the research of Yafi FA, et al., more than 80% of the cause of ED has an organic aetiology and, of them, the vasculogenic disorder is the most common and can involve arterial inflow disorders and abnormalities of venous outflow (corporeal veno‐occlusion) of corpus cavernosum.^4^ Since 1998, a couple of phosphodiesterase type 5 inhibitor (PDE5I) have been available as oral medication of ED. However, novel therapies are long expected to be developed because of the risks of PDE5I including rare but serious adverse effect of myocardial infarction and the fact that these conventional treatments were only used as transient care to sustain enough erection during a sexual intercourse. Recently, human umbilical cord blood stem cells have shown beneficial effects on erectile function when administered into the penises of men with severe type 2 diabetes.^5^ However, this effect was short‐lived and not durable. In the meantime, in basic research of the field of regenerative medicine worldwide, a number of papers have shown that bioactive substances secreted from stem cells *in vitro* are more important for tissue regeneration than stem cells themselves.^6^, ^7^, ^8^, ^9^ We have recently conducted a clinical pilot study of regenerative medicine on androgenic alopecia using a stem cell‐derived growth factor, which is produced from the components of conditioned media where stem cells derived from the exfoliated deciduous dental pulp (SHED‐CM) during the transformation of deciduous teeth into permanent teeth is cultured (manuscript in submission). In this clinical study, with the aim of establishing preemptive self‐regenerative medicine, we investigated whether the recovery of sexual function can be achieved through the cellular regeneration of damaged vascular tissue in the corpus cavernosum of the penis by direct SHED‐CM injection.

## 2 SUBJECTS, MATERIALS AND METHODS

### 2.1 Isolation of stem cells from human exfoliated deciduous teeth (SHED)

SHED were isolated as previously described.^10^ Briefly, exfoliated deciduous teeth (from 6‐ to 12‐year‐old individuals) were collected. Written informed consent was provided from each patients' guardians. Subsequent to separation of the crown and root, the dental pulp was isolated and then digested in a solution of 3 mg/mL collagenase type I (Worthington Biochem) and 4 mg/mL dispase (Roche Diagnostic/Boehringer Mannheim Corp.) for 1 h at 37°C. Single‐cell suspensions (1 × to 2 × 10^4^ cells/mL) were plated on culture dishes containing Dulbecco's modified Eagle medium (DMEM, Sigma‐Aldrich) supplemented with 10% foetal calf serum (FCS, Sigma‐Aldrich) at 37°C in 5% CO_2_ condition. An obtained cell population was designated as SHED. When the cells proliferated to 80–100% confluency, they were passaged using TrypLE™ Select (Gibco, Thermo Fisher Scientific) and passaged until P4. All P4 passaged cells were further used for collecting conditioned medium (CM).

### 2.2 SHED characterization

SHED were characterized as expressing a set of mesenchymal stem cell markers (ie, CD90, CD73 and CD105), but not endothelial/haematopoietic markers (ie, CD45, CD34, CD11b and HLA‐DR) using flow cytometry analysis.^7^ SHED exhibited adipogenic, chondrogenic and osteogenic differentiation as previously described.^10^

### 2.3 Preparation of SHED‐CM

SHED were cultured up to the order of 1 × 10^7^. At the time of confluence in the flask under sufficient cultivation, a medium containing serum was aspirated and switched to fresh serum‐free DMEM. After 48 h, the CM was collected and centrifuged at 2000 × *g* for 10 min at 4°C. To thoroughly remove cellular components, the supernatant was filtered through a 0.22‐micrometre filter and confirmed microscopically that there are no residual cells in the medium. As a quality control for a good manufacturing practice (GMP) grade clinical use, endotoxin test as well as bacterial test was performed. The concentration of CD63‐positive, 50–150 nm exosomes in the SHED‐CM was measured as 2.3 × 10^9^ particles/mL (data not shown).

### 2.4 Patients

A total of 38 Japanese patients were enrolled in this study who visited Ginza Solaria Clinic for the purpose of ED treatment during the study period starting April 2016 and ending October 2020.

### 2.5 Exclusion criteria

Any patients who were under treatment with other medication, such as PDE5I for ED or testosterone replacement therapy for late‐onset hypogonadism, were excluded from the study.

### 2.6 Study protocol

SHED‐CM was injected directly into the penis; briefly, a small soft hairband is loosely attached to the base of the penis, and 2 cc of SHED‐CM is directly injected once into each of right and left corpus cavernosum using an ultrafine needle. Loosely attached hairband was deliberately placed for 6 h mainly for the purpose of remaining patient's caution to maintain a rest care of injected site rather than securing the prolonged retention of SHED‐CM. Essential treatment was comprised of a total of 3 SHED‐CM injections at a week interval. Treatment could be extended to a maximum of consecutive 8 injections if a certain favourable outcome was obtained but still judged that more improvement was desired by the patient.

### 2.7 Clinical assessment

Efficacy of the treatment was assessed using the standard questionnaire of International Index of Erectile Function (IIEF‐5, validated Japanese version), which is clinically used to screen for ED and to assess treatment efficacy.^11^

### 2.8 Ethics statement

This clinical study was approved by the Internal Ethics Review Committee (Approval No. *Solaria 2016–001*) and carried out in accordance with the Declaration of Helsinki, and all the enrolled patients provided informed consent before participating in the current study.

### 2.9 Data analysis

A Mann‐Whitney *U* test or Wilcoxon signed‐rank test was used to compare changes in pretreatment and posttreatment clinical values. All statistical tests were performed using SPSS Statistics Ver. 25 (IBM Corporation), and *p* < 0.05 was considered statistically significant.

## 3 RESULTS

### 3.1 Patient characteristics

The average age and initial IIEF‐5 score of the 38 patients enrolled in this study was 56 (range: 31–79) years old and 13.1 (5–20) points, respectively (Table 1). Pretreatment serum total testosterone level was available in 14 patients, and the average was 5.36 (1.54–12.30) ng/mL. Medical history revealed 7 patients with diabetes, 7 patients with hypertension and 1 patient with priapism undergone shunt operation 6 months prior to the presentation to our clinic. Thirty‐two patients (84.2%) were treated with a total of 3 courses of SHED‐CM therapy and 3 patients with 5 courses, 2 patients with maximum of 8 courses. Rest of 1 patient was treated with a single treatment.

**TABLE 1:** Characteristics of patients enrolled in the study

	Pretreatment	Treatment outcomes		*p*‐value**
	Overall	IIEF‐5 21<	IIEF‐5 ≤21	
*n*	38	18	20	
Age (year)				
Average	56	51	60	**0.023**
Range	31–79	31–75	36–79	
History of DM/HT/priapism				
*n*	13	2	11	**0.012**
%	41.9	15.4	61.1	
Pre‐IIEF‐5 score (point)				
Average	13.1	17.1	9.5	**<0.0001**
Range	5–20	13–20	5–16	
Testosterone (ng/ml)*				
Average	5.36	5.96	4.76	0.949
Range	1.54–12.30	2.95–12.30	1.54–6.95	
SHED‐CM treatment course #				
Average	3.4	3.1	3.6	0.394
Range	1–8	3–5	1–8	

* 14 patients (IIEF‐5 >21; 7 patients, IIEF‐5 ≤21; 7 patients). Bolded red value are significant.

** Mann‐Whitney *U* test. *p* < 0.05 is defined statistically significant.

### 3.2 Clinical outcomes

**FIGURE 1:**
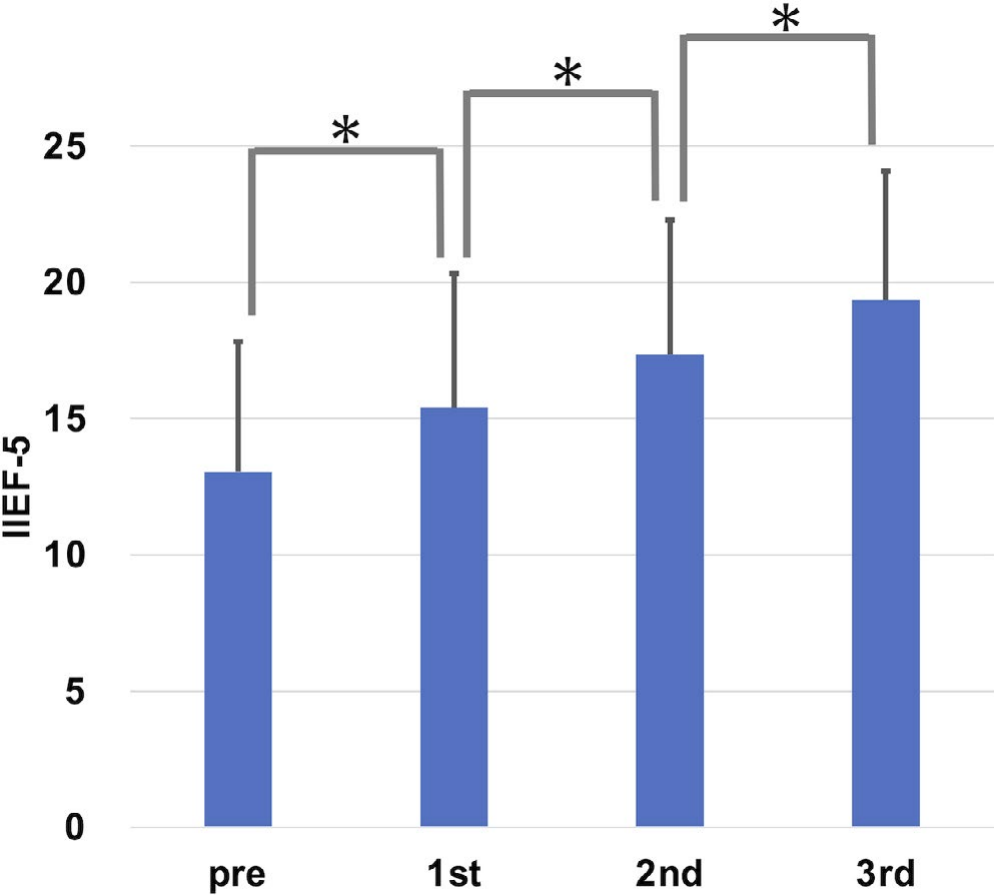
IIEF‐5 score between each treatment interval until the 3rd injection in overall patients. *p < 0.0001 (Wilcoxon signed‐rank test)

**FIGURE 2:**
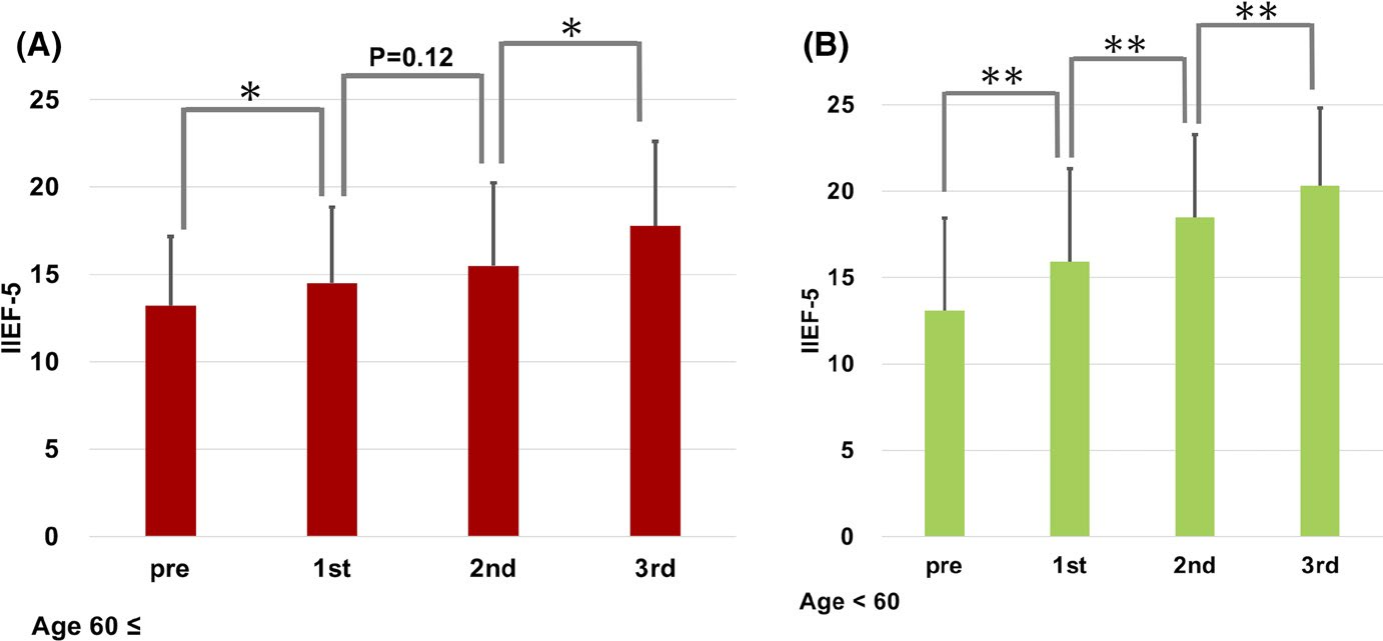
IIEF‐5 score between each treatment interval until the 3rd injection in the group of patients with ages 60≤ (A) and ages <60 (B). *p < 0.01, **p < 0.0001 (Wilcoxon signed‐rank test)

Thirty‐seven patients (97.4%) showed a statistically significant improvement of IIEF‐5 score with an average of 19.3 (7–25) points or 64.4 (10.0–300.0) % increase after three courses of SHED‐CM treatment (*p* < 0.0001, Wilcoxon signed‐rank test). There were significant incremental increases of IIEF‐5 between each treatment interval until the 3rd injection (Figure 1). This observation was consistent when the data were sub‐analysed by age (Figure 2). Until the last SHED‐CM therapy, 18 patients (47.4%) achieved more than 21 points in IIEF‐5 (no ED) and the plateau level of efficacy was maintained in all of these 18 patients until the end of the observation period (Table 2).

**TABLE 2:** Changes in IIEF‐5 score by SHED‐CM therapy

Case#	Age	IIEF‐5 score								
		Number of SHED‐CM therapy								
		pre	1st	2nd	3^rd^	4th	5th	6th	7th	8th
**22**	68	5	5	5	7	9	11			
**27**	42	5	5	7	8	8	11	13	18	20
**15**	58	12	12	12	12					
**19**	74	8	10	12	13					
**28**	79	12	14	14	14	14	14	14	17	17
**3**	59	7	12	12	14					
**29**	65	12	13	14	15					
**30**	59	8	9	12	16					
**36**	45	9	12	13	16					
**18**	61	13	15	13	16					
**14**	73	11	11	16	16					
**26**	73	11	11	11	17	15	17			
**12**	53	5	5	13	18					
**7**	55	8	11	15	18					
**20**	72	14	16	14	19					
**4**	61	16	17	17	19					
**17**	36	6	10	18	19					
**13**	71	12	14	16	20					
**34**	42	5	14	18	20					
**31**	52	13	17	20	22					
**32**	51	13	17	20	22					
**10**	52	20	21	20	22					
**23**	75	19	20	21	22					
**35**	57	16	21	22	22					
**11**	68	17	17	21	23					
**1**	53	16	21	21	23					
**24**	65	17	21	21	23	25	25			
**2**	54	18	20	22	23					
**37**	54	17	21	23	23					
**33**	31	18	22	23	23					
**6**	40	14	17	21	24					
**16**	45	19	18	22	24					
**9**	32	16	19	22	24					
**8**	31	16	20	22	24					
**5**	67	18	19	22	25					
**25**	39	20	21	22	25					
**38**	51	20	21	25	25					
**21**	64	10	16							

			**1−7**	**Severe**
				
			**8−11**	**Moderate**
				
			**12−16**	**Mild to moderate**
				
			**17−21**	**Mild**
				
			**22−25**	**No ED**
				

### 3.3 Characteristics of responding patients

These 18 patients with more than 21 points in IIEF‐5 (no ED) after SHED‐CM therapy were significantly younger in age and higher in pretreatment IIEF‐5 score than the rest of 20 patients with less or equal to 21 points in IIEF‐5 (average 51 vs 60 years old, *p* = 0.023; average 17.1 vs 9.5 points, *p* < 0.0001, respectively; Table 1). Also, these 18 responding patients with more than 21 points in IIEF‐5 (no ED) had significantly less medical history of diabetes, hypertension or priapism (15.4% vs 61.1%, *p* = 0.012). Although pretreatment serum testosterone level was higher in the responding patients, no statistically significant difference was observed between two groups (average 5.96 vs 4.76 ng/mL, *p* = 0.949). Of 20 patients with less or equal to 21 points in IIEF‐5, 4 patients extended up to 8 SHED‐CM injections to pursue better results. All of 4 patients achieved average of 129.0 (41.7–300.0) % increase from the pretreatment level in IIEF‐5 score after the end of extended SHED‐CM therapy (Table 1).

### 3.4 Adverse events

No adverse events or technical complications were encountered during SHED‐CM administration and entire follow‐up periods up to the present.

## 4 DISCUSSION

Research into regenerative medicine, in which stem cells including induced pluripotent stem (iPS) cells and embryonic stem (ES) cells are transplanted, is drawing attention as a new treatment for central nervous system diseases such as cerebral infarction and spinal cord injury.^12^, ^13^ However, there are many problems with stem cell transplantation, such as hazardous immune rejection against allografts and potential oncogenetic risk as well as complicating process of preservation and culture methods of iPS/ES cells.^14^, ^15^, ^16^ Recently, various studies have demonstrated that culture supernatants of stem cells possess ability of repairing damaged tissues, protect tissues, and ultimately regenerate organs with utilizing their own stem cells *in situ*.^17^ As such, the use of culture supernatant of stem cells is expected to be a next‐generation therapy in the field of regenerative medicine, which had been once thought to take a long time to translate into clinical application.

Our SHED‐CM treatment is characterized by the use of stem cells from rapidly differentiating deciduous dental pulp tissue, all of which are formulated in our hospital biolaboratory under a GMP grade quality control. SHED‐CM is a generic therapeutic agent that is generated directly from a conditioned medium, in which liquid components are produced by the culturing stem cells. Originally, SHED‐CM therapy was developed based on a research by Ueda M, et al.^18^ CM was originally made from stem cells of bone marrow, fat and umbilical cord. Recently, it has been reported that the CM of stem cells from deciduous or permanent dental pulps contains a large amount of proteins and cytokines.^8^, ^19^

Up to now, we have been conducting various preclinical trials on regenerative medicine using SHED‐CM. The combination of microneedle radiofrequency therapy and SHED‐CM has been shown to have an effect on skin wrinkles, pores and skin texture based on the activation of skin fibroblast collagen, elastin and hyaluronic acid production. Another study with systemic intravenous infusion of SHED‐CM showed reduction of the risk of cerebral infarction and myocardial infarction by preventing the occurrence of vascular endothelial cell damage as assessed by blood LOX‐index (R).^20^ Moreover, we have reported that patients with various paralysis after cerebral haemorrhage or cerebral infarction, who did not make improvements after 2 years of rehabilitation, showed remarkable improvements in motor disorders by nasal drip administration of SHED‐CM (Unpublished data). So far, concerns regarding safety issue generated by the fact that SHED‐CM contains a large number of biological components is not proven by the above previous clinical studies; however, we should continue carefully keeping this aspect in mind for the future direction.

It has been generally realized that the changes of physiological condition with ageing such as ED are difficult to be completely restored because certain irreversible pathological changes have already occurred in preceding asymptomatic stages before the clinical presentation of the disorder. However, in recent years, biomarkers of disease progression, such as individual genes, mRNAs, proteins, cellular metabolites and state‐of‐the‐art imaging studies, can be used to predict disease onset before disease are apparent. We believe that prophylactic treatment using SHED‐CM will lead to preemptive medicine to delay the onset of various diseases in the near future.

In this study, we examined whether SHED‐CM injection into the penis regenerates damaged vascular tissue in the corpus cavernosum of the penis at the cellular level and restores erectile function. From the results of our current study, patients with ED caused by mild‐to‐moderate vascular disorders appeared to be the proper candidates for the current SHED‐CM therapy.

In the process of erection, nitric oxide (NO) is released from the vascular endothelium of the corpus cavernosum of the penis to produce cyclic GMP, which subsequently dilates blood vessels. However, when endothelial cells are damaged, production of NO from the injured endothelial cells decreases. We speculate SHED‐CM may restore vasodilation of the corpus cavernosum by repairing the damaged vascular endothelial cells, which leads to an increased production of NO from endothelia.

With the evaluation of IIEF‐5, ED was defined when a total score was 21 points or less. In this study, an average 6.0 points (64.4%) increase was obtained, and consequently, IIEF‐5 score improved from an average of 13.1 points before treatment to an average of 19.3 points after three courses of SHED‐CM direct injection therapy (*p* < 0.0001). These 18 patients with more than 21 points in IIEF‐5 (no ED) at the end of the SHED‐CM therapy were significantly younger in age, higher in pretreatment IIEF‐5 score and had less preceding medical history which would be the cause of vascular complications than the rest of 20 patients with less responding patients.

As for the safety issues, we have encountered no adverse events during treatment period and whole through the follow‐up afterwards.

This study has some limitations. First of all, IIEF‐5 scores are based on the patients' subjective understanding of their own sexual status. However, this bias could be justified by the fact that most of the previous clinical studies in this field were also conducted with taking this score into account.^5^, ^21^ Second, we did not perform a pathological evaluation for accurately judging the effects of SHED‐CM to vascular endothelial cells. We believe that previous studies of SHED‐CM regarding the restoration of vascular damage might complement this aspect.^22^ Lastly, a significant limitation of this study is the lack of a control group. It is strictly limited for us to perform a randomized controlled study at our size of facilities, and therefore, we should take into account false‐positive results due to the placebo effect. Yet, our current clinical study has a clear advantage over previous clinical reports using allogeneic stem cells for ED treatment.^5^, ^21^

## 5 CONCLUSION

This is the first clinical report of ED treatment in the literatures with the use of SHED‐CM. Treatment with SHED‐CM is expected to repair vascular damages of the corpus cavernosum, which are the main cause of ED, and to be widely spread as a fundamental clinical application for ED patients.

## CONFLICT OF INTEREST

The authors confirm that there are no conflicts of interest.

## AUTHOR CONTRIBUTIONS

**Shoji Koga:** Conceptualization (lead); Data curation (equal); Formal analysis (equal); Funding acquisition (lead); Investigation (equal); Methodology (lead); Project administration (lead); Resources (lead); Software (equal); Supervision (lead); Validation (equal); Visualization (equal); Writing – original draft (supporting); Writing – review & editing (supporting). **Yutaka Horiguchi:** Conceptualization (supporting); Data curation (equal); Formal analysis (equal); Funding acquisition (supporting); Investigation (equal); Methodology (equal); Project administration (supporting); Resources (supporting); Software (equal); Supervision (supporting); Validation (equal); Visualization (equal); Writing – original draft (lead); Writing – review & editing (lead).
